# Bran data of total flavonoid and total phenolic contents, oxygen radical absorbance capacity, and profiles of proanthocyanidins and whole grain physical traits of 32 red and purple rice varieties^☆^

**DOI:** 10.1016/j.dib.2016.05.001

**Published:** 2016-05-10

**Authors:** Ming-Hsuan Chen, Anna M. McClung, Christine J. Bergman

**Affiliations:** a Agricultural Research Service, United States Department of Agriculture, Dale Bumpers National Rice Research Center, Stuttgart, AR 72160, USA; b Department of Food and Beverage, University of Nevada-Las Vegas, Las Vegas, NV 89154, USA

**Keywords:** Purple rice, Red rice, Black rice, Proanthocyanidins, Tannins, Flavonoids, Rice bran

## Abstract

Phytochemicals in red and purple bran rice have potential health benefit to humans. We determined the phytochemicals in brans of 32 red and purple global rice varieties. The description of the origin and physical traits of the whole grain (color, length, width, thickness and 100-kernel weight) of this germplasm collection are provided along with data of total flavonoid and total phenolic contents, oxygen radical absorbance capacity and total proanthocyanidin contents. The contents and proportions of individual oligomers, from degree of polymerization of monomers to 14-mers, and polymers in bran of these 32 rice varieties are presented (DOI: http://dx.doi.org/10.1016/j.foodchem.2016.04.004) [1].

**Specifications table**

**Table:** 

Subject area	Chemistry
More specific subject area	Phytochemicals
Type of data	Table
How data was acquired	Total flavonoid and total phenolic contents were determined using colorimetric methods. Profiling of proanthocyanidins and total proanthocyanidin content were determined using HPLC-fluorescence detection. Color of whole grain was measured in the CIE L*a*b* color space using a colorimeter. Length and width of whole grain rice were determined using an image analysis system. Thickness of whole grain was measured with a Digimatic indicator.
Data format	Analyzed
Experimental factors	Phytochemicals were determined in bran after solvent extraction.
Experimental features	The extractable total flavonoid and total phenolic contents, antiradical capacity, and oligomers and polymers of proanthocyanidins in bran of red and purple rice were determined.
Data source location	Beaumont, TX, USA
Data accessibility	The data are with this article.

**Value of the data**

• A large collection of globally diverse germplasm was selected for the phytochemical analysis of red and purple rice bran. Our data has the widest range of proanthocyanidins concentration reported in red rice bran thus far. These rice genotypes can be used to study genetics that control the synthesis of proanthocyanidins.
• First report of the contents and proportions of oligomers and polymers of proanthocyanidins in rice bran using HPLC-fluorescence detection.
• Genotypes differed significantly for phytochemicals found in the bran layer, indicating variation in the biosynthesis of these compounds. The colorimeter may be used to predict presence of some phytochemical in whole grain rice during varietal development.
• Genotypes were identified that have different phenolic profiles. They will be suitable for further study of the potential health benefits of these particular profiles.

## 1 Data

A detailed description of this germplasm collection (28 red and 4 purple bran rice varieties) is provided in Table 1, which includes the global origin and whole grain physical traits. Data in Table 2 includes the contents of total flavonoid, total phenolic, and total proanthocyanidins, along with oxygen radical absorbance capacity (ORAC). Data in Table 3 presents the content of individual oligomers (degree of polymerization of monomer to 14-mer) and polymers of proanthocyanidins. Table 4 provides data of the proportions of individual oligomers and polymers of proanthocyanidins.

## 2 Experimental design, materials and methods

### 2.1 Rice varieties and physical traits

The 32 rice varieties were obtained from the USDA National Small Grain Collection. The origin, accession numbers, and visual bran color of these 32 varieties are provided in Table 1. The rice varieties were grown under field conditions in 2009 and 2010 in Beaumont, TX using standard management practices for the area [1]. The color of whole grain rice was measured in the CIE L^*^a*b* color space using a colorimeter as described in [1] (Table 1). The length, width, thickness and 100-kernel weight of whole grain rice were determined as described in [2] (Table 1).

### 2.2 Sample preparation and extraction

The rough rice was dehulled and milled to collect the bran [1]. The bran was extracted with acetone/water/ acetic acid (70/29.5/0.5 (v/v/v)) as described in [1]. The sample extract was used for the determination of total flavonoid, total phenolic, ORAC and proanthocyanidin profiling as described in [1].

## 3 Total flavonoid, total phenolic and ORAC determination

Total flavonoid, total phenolic and ORAC in sample extracts were determined according to the methods in [1].

## 4 Profiling of proanthocyanidins

The sample extract was first purified through a LH-20 column prior to HPLC analysis as described in [1]. Proanthocyanidins were resolved on a HPLC-Diol column, detected by fluorescence detection and the concentration of individual oligomers (ranged from monomer to 14-mer) and polymers were determined using standards purified from cocoa (*Theobroma cacao*) as described in [3]. The total proanthocyanidin concentration was the sum of concentrations of all oligomers and polymers.

## Figures and Tables

**Table 1:** Origin and whole grain physical trait^1^ data of the 32 red and purple bran rice accessions.

ID	Origin	Accession no.	Bran color-GRIN^2^	Bran color-BMT^3^	L*	a*	b*	Chroma	Hue angle	100-kernel weight(g)	Length (mm)	Width(mm)	Thickness (mm)
242	Taiwan	PI 245710	Purple	Purple	31.28±3.58	11.81±0.39	12.76±0.01	17.39±0.26	47.22±.98	2.32±0.23	6.34±0.07	2.80±0.05	1.91±0.04
516	Uruguay	PI 400273	Red	Red	46.75±2.11	15.99±0.32	26.58±0.60	31.02±0.68	58.97±0.06	2.67±0.14	6.37±0.20	2.88±0.11	2.13±0.08
Banjul	Gambia	PI 414546	Red	Red	41.37±0.36	15.08±0.12	23.53±0.34	27.97±0.37	57.37±0.21	2.29±0.04	6.07±0.07	2.72±0.02	1.92±0.00
Baros	Guyana	PI 184386	Red	Red	44.74±1.95	13.45±2.07	22.58±2.09	26.28±2.85	59.31±1.55	2.10±0.07	6.82±0.06	2.54±0.04	1.76±0.03
Choko To	China	PI 389319	Red	Red	44.72±2.05	13.75±1.73	24.12±2.06	27.76±2.65	60.35±1.01	3.64±0.38	8.89±0.51	2.49±0.07	2.14±0.03
D16	United States	PI 506229	Red	Red	34.29±1.90	14.84±0.80	19.02±2.02	24.13±2.09	51.97±1.47	1.51±0.15	5.60±0.31	2.28±0.04	1.69±0.00
DJ 24	Bangladesh	PI 403082	Red	Red	46.48±2.79	13.93±0.16	24.94±1.80	28.57±1.50	60.76±2.04	2.01±0.07	5.44±0.04	2.77±0.01	1.94±0.08
DM 77	Bangladesh	PI 403173	Red	Red	48.93±3.31	14.41±0.70	26.86±1.59	30.48±1.73	61.79±0.26	2.46±0.28	5.82±0.11	2.90±0.05	2.05±0.05
GPNO 28809	Uncertain	PI 510190	Red	Red	45.18±0.35	13.58±1.60	23.78±1.47	27.39±2.07	60.31±1.39	2.27±0.35	6.43±0.37	2.69±0.12	1.90±0.07
IARI 5828	India	PI 353648	Red	Red	39.12±0.87	15.82±0.05	23.92±0.86	28.69±0.69	56.50±1.03	2.05±0.05	5.06±0.06	3.08±0.03	2.07±0.07
IARI 6627	India	PI 353724	Red	Red	50.80±4.07	11.41±1.63	25.34±0.56	27.81±1.18	65.82±2.59	1.39±0.00	4.92±0.09	2.46±0.06	1.73±0.03
IITA 119	Nigeria	PI 458466	Red	Red	42.85±0.16	14.83±0.04	22.49±0.13	26.94±0.13	56.60±0.08	2.31±0.17	6.47±0.27	2.64±0.07	1.90±0.02
Unknown^4^	unknown	PI 400798	Purple	Purple	28.93±0.85	12.76±0.68	13.58±0.98	18.63±1.18	46.77±0.55	2.39±0.09	6.26±0.19	2.87±0.01	1.89±0.05
IL 121-1-1	USA	breeding line	Red	Red	36.54±2.35	14.17±0.44	19.78±1.48	24.35±0.94	54.33±2.88	2.11±0.00	6.77±0.28	2.31±0.02	1.86±0.07
Kakani 2	Nepal	PI 400020	Light Brown	Red	47.38±0.35	9.22±0.55	20.85±0.29	22.80±0.49	66.15±0.96	2.09±0.25	5.48±0.28	2.90±0.15	1.99±0.02
Kun Shan Wu Shan Keng	Taiwan	PI 282941	Red	Red	42.00±4.25	11.12±1.91	21.65±0.07	24.37±0.81	62.88±4.06	2.19±0.40	5.59±0.25	2.91±0.22	2.09±0.10
Malagkit Pirurutong	Philippines	PI 434618	Purple	Purple	27.49±0.28	12.82±1.52	12.78±1.00	18.10±1.78	44.98±1.17	2.38±0.06	6.22±0.20	2.80±0.00	1.90±0.03
Manzano	Zaire	PI 430394	Red	Red	42.52±1.22	15.23±0.57	24.50±0.28	28.85±0.06	58.14±1.26	2.69±0.10	7.12±0.16	2.73±0.07	1.87±0.04
Muong Pra	Vietnam	PI 392745	Brown	Red	41.56±1.42	12.08±1.53	23.11±0.66	26.11±0.12	62.41±3.64	1.54±0.01	5.73±0.11	2.23±0.01	1.77±0.07
Neang Meas	Cambodia	PI 389951	Red	Red	46.11±1.98	16.68±0.50	27.12±1.08	31.84±0.65	58.39±1.79	2.75±0.15	6.28±0.14	3.09±0.07	2.06±0.11
Ngasein	Myanmar	PI 392577	Red	Red	50.34±3.36	11.59±0.31	28.59±3.38	30.87±3.01	67.79±2.90	1.84±0.01	5.25±0.07	2.73±0.01	1.95±0.05
P 1293	Turkey	PI 431343	Red	Red	40.75±3.89	15.73±0.75	24.10±1.96	28.80±1.23	56.79±3.37	1.68±0.03	5.83±0.14	2.33±0.03	1.72±0.06
Paray Kinta Kabaras	Philippines	PI 392997	Purple	Purple	27.91±0.01	9.21±0.97	10.68±1.51	14.10±1.78	49.15±1.03	2.42±0.11	5.81±0.21	3.05±0.03	1.98±0.05
Popey	Cambodia	PI 392610	Red	Red	45.43±1.85	14.82±3.57	25.35±3.17	29.38±4.54	59.92±2.92	1.48±0.07	5.49±0.19	2.36±0.08	1.64±0.01
Red Wells	USA	breeding line	Red	Red	35.53±2.11	14.60±0.78	19.75±2.85	24.58±2.76	53.38±2.50	2.28±0.06	7.59±0.02	2.18±0.01	1.84±0.07
Samanoek	Indonesia	PI 389872	Red	Red	43.28±2.71	15.25±1.17	23.88±1.28	28.36±0.45	57.41±3.39	2.70±0.12	6.49±0.02	2.88±0.01	1.98±0.02
SL 22-633	Sierra Leone	PI 433804	Red	Red	40.84±0.56	12.72±0.78	21.86±0.23	25.29±0.59	59.82±1.27	1.93±0.14	6.04±0.23	2.66±0.07	1.73±0.01
SL 31-709	Sierra Leone	PI 433823	Red	Red	46.34±2.56	13.03±0.46	24.25±0.07	27.53±0.16	61.75±0.92	2.64±0.29	6.94±0.44	2.81±0.12	1.83±0.11
Tieu Dong^5^	Vietnam	PI 392788	Red	Red	38.51±2.19	15.78±0.05	21.85±1.21	26.96±1.01	54.14±1.43	1.84±0.00	5.68±0.10	2.54±0.00	1.82±0.01
Timonchette 3	Haiti	CIor 8915	Red	Red	42.53±1.19	16.96±0.94	25.57±0.07	30.69±0.46	56.45±1.54	2.24±0.01	6.16±0.06	2.67±0.02	1.90±0.05
WC 10346	Uncertain	PI 469393	Brown	Red	50.29±1.55	13.03±0.34	25.94±0.36	29.03±0.17	63.33±0.91	2.11±0.22	6.54±0.23	2.64±0.13	1.79±0.04
Yanayanan	Philippines	PI 373179	Red	Red	42.92±3.71	14.51±2.78	23.20±0.67	27.40±2.04	58.14±4.20	2.03±0.02	6.37±0.19	2.54±0.02	1.75±0.03

1 Mean±1 standard deviation of 2009 and 2010 data; Color parameters: L*, lightness; a*, redness; b*, yellowness; Chroma, color intensity.

2 Bran color, based on GRIN (Germplasm Resources Information Network) (http://www.ars-grin.gov/cgi-bin/npgs/html/desc.pl?75011).

3 Bran color observation by authors.

4 Listed as light brown bran color for accession PI 400798 in GRIN; received seeds were purple bran.

5 Tieu Gong, listed as both light brown and red in GRIN, only red bran line was used for the study.

**Table 2:** Data^1^ of total flavanoids (TF), total phenolics (TP), oxygen radical absorbance capacity (ORAC) and total proanthocyanidin (TPA) in bran of 32 red and purple rice accessions.

ID^2^	TF	TP	ORAC	TPA
242	28.0±0.3	70.1±3.4	626.6±74.5	24.60±1.46
516	13.2±0.4	37.9±3.9	304.8±23.5	13.07±1.64
Banjul	22.0±1.1	57.3±1.8	509.6±31.4	20.17±0.81
Baros	20.5±3.1	53.5±7.8	492.2±8.1	23.02±10.77
Choko To	21.5±1.5	57.1±4.0	377.2±98.5	21.79±2.85
D16	18.8±1.5	51.1±6.7	384.2±68.3	12.74±3.10
DJ 24	17.7±3.1	47.0±5.3	407.4±46.4	18.11±5.58
DM 77	10.3±1.6	28.3±3.5	314.0±144.2	9.50±0.81
GPNO 28809	18.2±0.5	45.9±0.2	447.2±50.6	20.37±2.18
IARI 5828	18.3±1.2	52.3±0.4	368.7±84.0	17.39±0.55
IARI 6627	24.2±0.9	61.5±1.7	757.0±247.4	30.37±2.35
IITA 119	20.0±1.5	54.1±4.2	283.9±23.3	14.83±3.61
Unknown^2^	26.8±1.3	67.9±5.1	759.8±27.5	22.31±2.36
IL 121-1-1	15.6±3.5	41.8±10.8	353.1±112.6	12.03±5.60
Kakani 2	23.2±3.3	59.0±12.8	702.9±41.2	36.15±10.36
Kun Shan Wu Shan Keng	21.0±5.4	51.2±10.8	517.6±154.3	26.21±0.05
Malagkit Pirurutong	27.8±1.2	70.0±4.2	731.8±11.2	20.84±3.24
Manzano	17.9±0.3	48.3±0.1	470.6±15.4	16.31±0.41
Muong Pra	19.1±0.7	51.0±1.6	449.4±130.4	20.65±1.60
Neang Meas	16.6±0.5	47.4±3.4	381.0±84.8	17.17±2.39
Ngasein	13.8±4.6	37.4±8.5	416.9±158.6	13.49±3.58
P 1293	16.1±3.7	43.2±8.2	382.5±67.5	11.93±4.75
Paray Kinta Kabaras	24.5±4.0	66.1±7.7	784.0±45.8	22.56±1.67
Popey	18.4±5.7	49.5±15.0	431.1±209.2	20.15±9.16
Red Wells	13.0±0.1	36.8±2.1	289.5±101.3	8.38±0.60
Samanoek	17.1±2.8	45.7±5.1	335.5±22.3	15.42±0.09
SL 22-633	21.0±2.8	57.4±6.6	474.5±139.5	19.29±1.12
SL 31-709	24.7±0.5	63.2±3.5	549.2±68.2	30.79±4.73
Tieu Dong^3^	17.1±1.2	45.8±3.4	301.7±85.3	11.23±3.42
Timonchette 3	15.3±3.1	42.0±5.4	341.4±33.5	10.89±4.73
WC 10346	23.3±0.7	58.4±3.5	465.0±183.5	30.22±1.19
Yanayanan	18.4±2.4	53.4±8.2	430.1±148.2	20.23±9.59

1 TF, mg catechin eq/g bran; TP, mg gallic acid eq/g bran; ORAC, oxygen radical absorbance capacity, µM Trolox eq/g bran; TPA, sum of monomers to polymers of proanthocyanidin concentration determined by HPLC-fluorescence detection, mg/g bran.

2 Detail information of accessions is listed in Table 1.

**Table 3:** Data^1^ of oligomer and polymer profiling of proanthocyanidins (mg/g) in bran of 32 red and purple rice accessions.

ID^2^	DP1	DP2	DP3	DP4	DP5	DP6	DP7	DP8	DP9	DP10	DP11to14	Polymer (DP > 14)	TPA^3^
242	0.13±0.03	0.52±0.10	1.17±0.15	1.40±0.13	1.46±0.02	1.64±0.03	1.79±0.05	1.43±0.04	1.68±0.14	1.82±0.10	4.98±0.27	6.59±0.41	24.60±1.46
516	0.05±0.01	0.25±0.05	0.70±0.00	0.78±0.08	0.79±0.03	0.85±0.01	0.91±0.05	0.71±0.05	0.84±0.11	0.92±0.14	2.63±0.54	3.63±0.90	13.07±1.64
Banjul	0.08±0.00	0.31±0.01	0.90±0.01	1.12±0.08	1.16±0.00	1.31±0.02	1.43±0.03	1.14±0.04	1.35±0.06	1.51±0.09	4.28±0.27	5.58±0.23	20.17±0.81
Baros	0.10±0.05	0.45±0.25	1.18±0.69	1.26±0.62	1.31±0.61	1.43±0.63	1.54±0.67	1.21±0.52	1.45±0.65	1.60±0.68	4.67±2.13	6.82±3.29	23.02±10.77
Choko To	0.07±0.03	0.24±0.13	0.71±0.20	0.74±0.28	0.89±0.26	1.09±0.26	1.27±0.27	1.08±0.22	1.33±0.25	1.58±0.29	5.05±0.92	7.76±0.27	21.79±2.85
D16	0.03±0.00	0.16±0.06	0.60±0.22	0.74±0.15	0.73±0.23	0.80±0.23	0.87±0.23	0.69±0.18	0.84±0.21	0.93±0.23	2.68±0.68	3.68±0.67	12.74±3.10
DJ 24	0.07±0.04	0.37±0.14	1.01±0.38	1.20±0.39	1.24±0.38	1.32±0.38	1.38±0.39	1.06±0.29	1.22±0.36	1.33±0.40	3.59±1.13	4.32±1.29	18.11±5.58
DM 77	0.04±0.01	0.18±0.01	0.51±0.06	0.63±0.09	0.62±0.04	0.65±0.04	0.67±0.04	0.52±0.03	0.60±0.04	0.68±0.07	1.96±0.19	2.43±0.19	9.50±0.81
GPNO 28809	0.13±0.07	0.53±0.18	1.35±0.31	1.43±0.33	1.46±0.32	1.53±0.28	1.57±0.23	1.20±0.14	1.34±0.10	1.47±0.09	3.94±0.11	4.43±0.02	20.37±2.18
IARI 5828	0.06±0.01	0.24±0.02	0.65±0.11	0.77±0.02	0.86±0.03	1.00±0.03	1.12±0.03	0.91±0.01	1.10±0.00	1.26±0.04	3.76±0.08	5.68±0.70	17.39±0.55
IARI 6627	0.13±0.03	0.63±0.16	1.63±0.35	2.07±0.25	2.11±0.16	2.29±0.13	2.41±0.12	1.89±0.08	2.10±0.06	2.29±0.07	6.30±0.04	6.51±0.98	30.37±2.35
IITA 119	0.02±0.00	0.10±0.01	0.35±0.11	0.47±0.09	0.61±0.12	0.75±0.16	0.90±0.21	0.76±0.18	0.96±0.25	1.12±0.31	3.61±1.06	5.17±1.10	14.83±3.61
Unknown	0.11±0.02	0.47±0.09	1.07±0.16	1.28±0.15	1.33±0.07	1.50±0.08	1.64±0.12	1.30±0.12	1.55±0.18	1.68±0.17	4.61±0.51	5.77±0.69	22.31±2.36
IL 121-1-1	0.03±0.02	0.16±0.11	0.43±0.29	0.54±0.32	0.58±0.36	0.67±0.39	0.76±0.43	0.62±0.34	0.75±0.40	0.86±0.45	2.64±1.28	4.00±1.21	12.03±5.60
Kakani 2	0.20±0.01	0.78±0.01	1.96±0.18	2.44±0.21	2.61±0.41	2.85±0.54	3.01±0.66	2.39±0.59	2.57±0.68	2.69±0.84	7.15±2.53	7.50±3.75	36.15±10.36
Kun Shan Wu Shan Keng	0.11±0.02	0.58±0.00	1.56±0.09	2.04±0.34	2.09±0.32	2.21±0.33	2.25±0.26	1.70±0.15	1.89±0.08	1.98±0.01	5.07±0.41	4.73±0.95	26.21±0.05
Malagkit Pirurutong	0.10±0.02	0.43±0.07	1.01±0.16	1.21±0.22	1.28±0.32	1.44±0.35	1.56±0.37	1.21±0.26	1.47±0.29	1.57±0.29	4.27±0.77	5.30±0.12	20.84±3.24
Manzano	0.05±0.01	0.20±0.02	0.53±0.02	0.70±0.02	0.80±0.01	0.93±0.01	1.04±0.02	0.85±0.03	1.05±0.06	1.18±0.06	3.53±0.18	5.45±0.14	16.31±0.41
Muong Pra	0.10±0.01	0.44±0.02	1.16±0.16	1.27±0.10	1.32±0.05	1.43±0.02	1.52±0.08	1.21±0.10	1.40±0.16	1.56±0.17	4.35±0.59	4.88±0.79	20.65±1.60
Neang Meas	0.05±0.01	0.23±0.05	0.68±0.17	0.82±0.05	0.80±0.15	0.91±0.17	1.04±0.18	0.87±0.16	1.08±0.15	1.24±0.22	3.85±0.69	5.60±0.40	17.17±2.39
Ngasein	0.08±0.00	0.36±0.04	0.91±0.13	1.19±0.09	1.06±0.17	1.05±0.20	1.05±0.23	0.78±0.20	0.90±0.22	0.95±0.28	2.45±0.79	2.71±1.21	13.49±3.58
P 1293	0.09±0.03	0.32±0.11	0.77±0.27	0.82±0.32	0.73±0.24	0.74±0.24	0.77±0.26	0.62±0.21	0.68±0.26	0.81±0.33	2.28±0.99	3.29±1.50	11.93±4.75
Paray Kinta Kabaras	0.09±0.01	0.44±0.09	1.04±0.20	1.35±0.17	1.45±0.24	1.63±0.24	1.74±0.23	1.39±0.23	1.53±0.03	1.65±0.14	4.43±0.19	5.82±0.09	22.56±1.67
Popey	0.07±0.03	0.40±0.18	1.10±0.50	1.29±0.40	1.25±0.54	1.33±0.59	1.41±0.63	1.11±0.49	1.31±0.61	1.46±0.69	4.21±2.09	5.19±2.39	20.15±9.16
Red Wells	0.02±0.01	0.10±0.04	0.32±0.04	0.37±0.09	0.41±0.08	0.48±0.08	0.53±0.07	0.43±0.06	0.53±0.04	0.60±0.05	1.78±0.06	2.81±0.02	8.38±0.60
Samanoek	0.03±0.00	0.22±0.01	0.69±0.07	0.83±0.05	0.84±0.06	0.92±0.06	1.00±0.06	0.80±0.05	0.97±0.04	1.12±0.01	3.29±0.06	4.70±0.27	15.42±0.09
SL 22-633	0.04±0.01	0.18±0.03	0.52±0.04	0.76±0.02	0.95±0.03	1.17±0.05	1.35±0.05	1.10±0.02	1.32±0.06	1.50±0.07	4.37±0.27	6.03±0.66	19.29±1.12
SL 31-709	0.10±0.03	0.47±0.14	1.27±0.39	1.49±0.18	1.71±0.28	1.95±0.29	2.16±0.32	1.72±0.30	2.06±0.32	2.35±0.36	6.85±1.22	8.66±0.89	30.79±4.73
Tieu Dong	0.03±0.01	0.12±0.04	0.39±0.09	0.47±0.19	0.53±0.15	0.63±0.18	0.72±0.21	0.58±0.16	0.71±0.21	0.82±0.25	2.49±0.76	3.75±1.17	11.23±3.42
Timonchette 3	0.04±0.01	0.17±0.04	0.47±0.14	0.62±0.25	0.60±0.20	0.66±0.22	0.72±0.26	0.59±0.21	0.70±0.32	0.80±0.36	2.35±1.17	3.17±1.55	10.89±4.73
WC 10346	0.15±0.07	0.64±0.24	1.62±0.45	1.89±0.51	1.97±0.43	2.12±0.35	2.25±0.24	1.75±0.12	2.01±0.04	2.23±0.03	6.26±0.34	7.33±0.96	30.22±1.19
Yanayanan	0.06±0.03	0.31±0.20	0.86±0.53	1.00±0.49	1.09±0.57	1.23±0.62	1.37±0.68	1.11±0.54	1.34±0.63	1.53±0.71	4.43±2.07	5.90±2.52	20.23±9.59

1 Mean±1 standard deviation of 2009 and 2010 data; DP, degree of polymerization of oligomers and polymers.

2 Detail information of accessions is listed in Table 1.

3 TPA, total concentration of proanthocyanidins, sum of individual DP of PAs determined by HPLC.

**Table 4:** Distribution1 (%, w/w) of individual degree of polymerization (DP) of proanthocyanidins over total proanthocyanidins (TPA).

ID^2^	DP1	DP2	DP3	DP4	DP5	DP6	DP7	DP8	DP9	DP10	DP11 to 14	Polymer (DP > 14)
242	0.53±0.11	2.10±0.27	4.74±0.33	5.68±0.21	5.93±0.26	6.68±0.28	7.28±0.24	5.81±0.20	6.82±0.15	7.39±0.04	20.25±0.12	26.79±0.08
516	0.41±0.15	1.95±0.62	5.37±0.64	6.06±1.37	6.13±0.97	6.57±0.75	7.01±0.47	5.42±0.28	6.40±0.05	7.07±0.15	20.03±1.61	27.58±3.44
Banjul	0.37±0.02	1.53±0.00	4.46±0.23	5.52±0.23	5.75±0.18	6.46±0.16	7.08±0.11	5.64±0.03	6.70±0.00	7.51±0.12	21.28±0.38	27.70±0.00
Baros	0.43±0.00	1.92±0.19	4.96±0.66	5.44±0.14	5.70±0.02	6.24±0.20	6.73±0.22	5.32±0.24	6.32±0.15	7.04±0.35	20.35±0.27	29.54±0.45
Choko To	0.31±0.09	1.05±0.47	3.25±0.52	3.34±0.84	4.05±0.68	4.95±0.56	5.78±0.48	4.91±0.37	6.06±0.36	7.21±0.38	23.11±1.20	35.99±5.94
D16	0.23±0.03	1.24±0.15	4.60±0.64	5.82±0.21	5.66±0.39	6.22±0.30	6.78±0.13	5.41±0.06	6.61±0.08	7.31±0.05	21.01±0.26	29.11±1.82
DJ 24	0.38±0.13	2.04±0.16	5.54±0.38	6.60±0.14	6.82±0.02	7.32±0.15	7.64±0.21	5.86±0.18	6.76±0.10	7.36±0.07	19.80±0.12	23.86±0.24
DM 77	0.38±0.02	1.87±0.03	5.35±0.15	6.66±0.42	6.56±0.09	6.81±0.16	7.08±0.20	5.52±0.13	6.37±0.09	7.18±0.12	20.59±0.19	25.64±0.20
GPNO 28809	0.63±0.29	2.57±0.59	6.57±0.81	6.99±0.87	7.12±0.79	7.46±0.55	7.68±0.33	5.88±0.07	6.60±0.23	7.23±0.33	19.42±1.52	21.85±2.23
IARI 5828	0.33±0.06	1.36±0.18	3.73±0.72	4.42±0.24	4.97±0.35	5.73±0.36	6.42±0.37	5.21±0.25	6.35±0.23	7.26±0.00	21.61±0.25	32.61±2.99
IARI 6627	0.43±0.08	2.06±0.37	5.35±0.73	6.82±0.29	6.95±0.01	7.56±0.16	7.93±0.23	6.22±0.21	6.92±0.33	7.57±0.35	20.83±1.75	21.36±1.58
IITA 119	0.17±0.04	0.70±0.10	2.37±0.19	3.20±0.15	4.11±0.20	5.10±0.16	6.05±0.09	5.10±0.00	6.47±0.14	7.54±0.27	24.16±1.25	35.02±1.12
Unknown	0.50±0.03	2.11±0.17	4.80±0.21	5.71±0.08	5.97±0.31	6.75±0.34	7.35±0.24	5.84±0.09	6.94±0.09	7.51±0.05	20.67±0.11	25.83±0.34
IL 121-1-1	0.28±0.03	1.26±0.35	3.36±0.84	4.33±0.68	4.59±0.85	5.35±0.79	6.12±0.74	5.04±0.45	6.13±0.45	7.08±0.41	21.80±0.46	34.67±6.05
Kakani 2	0.57±0.19	2.27±0.68	5.58±1.11	6.96±1.42	7.35±0.98	8.00±0.80	8.42±0.60	6.65±0.29	7.13±0.16	7.42±0.21	19.59±1.38	20.07±4.63
Kun Shan Wu Shan Keng	0.43±0.07	2.21±0.01	5.96±0.34	7.78±1.30	7.97±1.21	8.42±1.23	8.58±0.99	6.49±0.55	7.22±0.28	7.56±0.06	19.34±1.60	18.05±3.64
Malagkit Pirurutong	0.46±0.03	2.06±0.03	4.83±0.04	5.82±0.16	6.09±0.58	6.84±0.63	7.46±0.60	5.79±0.34	7.04±0.28	7.49±0.22	20.45±0.51	25.69±3.42
Manzano	0.32±0.04	1.20±0.16	3.25±0.22	4.31±0.21	4.89±0.18	5.70±0.10	6.39±0.03	5.21±0.04	6.41±0.22	7.22±0.16	21.66±0.55	33.42±0.02
Muong Pra	0.50±0.03	2.14±0.26	5.69±1.20	6.17±0.95	6.42±0.75	6.95±0.42	7.39±0.17	5.88±0.01	6.77±0.23	7.55±0.25	21.01±1.22	23.55±1.99
Neang Meas	0.29±0.05	1.36±0.08	3.93±0.43	4.79±0.40	4.63±0.22	5.30±0.24	6.03±0.24	5.03±0.21	6.30±0.03	7.18±0.27	22.38±0.92	32.78±2.23
Ngasein	0.61±0.13	2.74±0.42	6.83±0.83	9.09±1.77	7.96±0.84	7.88±0.58	7.82±0.34	5.75±0.04	6.70±0.16	7.01±0.21	18.01±1.10	19.60±3.79
P 1293	0.73±0.04	2.74±0.19	6.56±0.37	6.92±0.06	6.20±0.48	6.33±0.48	6.55±0.40	5.27±0.33	5.77±0.15	6.76±0.04	18.97±0.71	27.19±1.76
Paray Kinta Kabaras	0.41±0.02	1.94±0.24	4.58±0.55	5.97±0.30	6.43±0.61	7.19±0.51	7.68±0.43	6.15±0.59	6.79±0.38	7.31±0.07	19.66±0.61	25.90±2.33
Popey	0.35±0.01	1.96±0.02	5.47±0.00	6.66±1.05	6.26±0.15	6.61±0.09	7.00±0.07	5.54±0.07	6.51±0.07	7.24±0.14	20.67±1.00	25.73±0.18
Red Wells	0.25±0.09	1.15±0.37	3.79±0.18	4.38±0.81	4.92±0.58	5.66±0.56	6.34±0.41	5.11±0.38	6.30±0.07	7.13±0.05	21.33±0.81	33.63±2.70
Samanoek	0.22±0.01	1.43±0.05	4.47±0.40	5.39±0.33	5.44±0.35	5.96±0.36	6.51±0.32	5.17±0.27	6.31±0.20	7.27±0.00	21.35±0.29	30.49±1.93
SL 22-633	0.22±0.04	0.95±0.19	2.71±0.39	3.95±0.31	4.92±0.12	6.09±0.11	7.02±0.14	5.71±0.21	6.82±0.11	7.76±0.09	22.65±0.08	31.21±1.62
SL 31-709	0.32±0.06	1.51±0.23	4.06±0.66	4.84±0.14	5.55±0.04	6.33±0.03	7.00±0.04	5.59±0.10	6.68±0.02	7.63±0.00	22.22±0.54	28.25±1.44
Tieu Dong	0.24±0.01	1.06±0.07	3.47±0.25	4.13±0.39	4.72±0.09	5.62±0.12	6.43±0.12	5.19±0.13	6.37±0.08	7.29±0.01	22.14±0.05	33.34±0.28
Timonchette 3	0.37±0.10	1.62±0.29	4.46±0.66	5.72±0.16	5.66±0.66	6.23±0.69	6.76±0.55	5.49±0.46	6.39±0.19	7.29±0.12	21.24±1.50	28.77±1.75
WC 10346	0.50±0.22	2.12±0.71	5.32±1.29	6.22±1.45	6.49±1.16	6.99±0.88	7.42±0.51	5.80±0.17	6.67±0.13	7.39±0.19	20.76±1.94	24.32±4.13
Yanayanan	0.29±0.03	1.45±0.33	4.09±0.68	4.94±0.07	5.31±0.29	6.05±0.20	6.73±0.16	5.48±0.06	6.63±0.02	7.56±0.05	21.96±0.20	29.50±1.53

^1^Mean±1 standard deviation of 2009 and 2010 data; DP, degree of polymerization of oligomers and polymers.

2 Detail information of accessions is listed in Table 1.
