# Supplementary material for: Bran data of total flavonoid and total phenolic contents, oxygen radical absorbance capacity, and profiles of proanthocyanidins and whole grain physical traits of 32 red and purple rice varieties
Source: Data Brief. 2016 May 10;8:6–13. doi: 10.1016/j.dib.2016.05.001 (PMC4878786; doi:10.1016/j.dib.2016.05.001)
Supplement: Supplementary file 1 — Supplementary material [file mmc1.docx]

Conflicts of Interest: none.
